# Highly Efficient Intracellular Protein Delivery by Cationic Polyethyleneimine-Modified Gelatin Nanoparticles

**DOI:** 10.3390/ma11020301

**Published:** 2018-02-15

**Authors:** Ming-Ju Chou, Hsing-Yi Yu, Jui-Ching Hsia, Ying-Hou Chen, Tzu-Ting Hung, Hsiao-Mei Chao, Edward Chern, Yi-You Huang

**Affiliations:** 1 Institute of Biomedical Engineering, National Taiwan University, Taipei 10617, Taiwan; ymlululu@gmail.com (M.-J.C.); d01548022@ntu.edu.tw (Y.-H.C.); 2 niChe Lab for Stem Cell and Regenerative Medicine, Department of Biochemical Science and Technology, National Taiwan University, Taipei 10617, Taiwan; a3553883@gmail.com (H.-Y.Y.); philhsia14809@gmail.com (J.-C.H.); neggcandy@gmail.com (T.-T.H.); mei999@gmail.com (H.-M.C.); 3 Department of Pathology, Wan Fang Hospital, Taipei Medical University, Taipei 11696, Taiwan

**Keywords:** gelatin, nanoparticle, polyethyleneimine, intracellular protein delivery, cationic nanocarrier

## Abstract

Intracellular protein delivery may provide a safe and non-genome integrated strategy for targeting abnormal or specific cells for applications in cell reprogramming therapy. Thus, highly efficient intracellular functional protein delivery would be beneficial for protein drug discovery. In this study, we generated a cationic polyethyleneimine (PEI)-modified gelatin nanoparticle and evaluated its intracellular protein delivery ability in vitro and in vivo. The experimental results showed that the PEI-modified gelatin nanoparticle had a zeta potential of approximately +60 mV and the particle size was approximately 135 nm. The particle was stable at different biological pH values and temperatures and high protein loading efficiency was observed. The fluorescent image results revealed that large numbers of particles were taken up into the mammalian cells and escaped from the endosomes into the cytoplasm. In a mouse C26 cell-xenograft cancer model, particles accumulated in cancer cells. In conclusion, the PEI-modified gelatin particle may provide a biodegradable and highly efficient protein delivery system for use in regenerative medicine and cancer therapy.

## 1. Introduction

Intracellular functional proteins, such as specific transcriptional factors or epigenetic modulators, may trigger cell fate transition/cell reprogramming during development and pathophysiological processes. Recently, several studies showed that cell reprogramming may be a new approach for regenerative medicine and disease therapy. In 2006, Yamanaka et al. transduced the transcription factors Oct4, Sox2 Klf4, and c-Myc with a retroviral system to induce fibroblasts to change their cell fate into a pluripotent state and generated induced pluripotent stem cells [1]. Liver fibrosis is a chronic inflammatory disease and severe liver fibrosis may lead toward liver cancer. Liver fibrosis is caused by hepatic stellate cell activation and extracellular matrix accumulation in the liver. The hepatic-specific transcription factors FOXA3, HNF1A, and HNF4A were reported to direct conversion of activated hepatic stellate cells into hepatocytes to reduce liver fibrosis [2]. In cancer differentiation therapy, cancer stem cells were converted into differentiated progeny cancer cells to increase the drug sensitivity and reduce the aggressiveness of cancer cells [3,4,5]. Thus, altering the expression of functional proteins in target cells may be applicable for the disease treatment and cancer cell differentiation therapy [6].

Cellular reprogramming factors can be expressed in target cells using DNA-viral systems for delivery, such as adenovirus, adeno-associated virus, and retrovirus [7]. However, DNA integration, oncogenicity, inflammatory reaction, and other toxic side effects are critical issues when using these viral systems. Thus, directly delivering cellular reprogramming proteins to target cells may be a safe strategy for clinical therapies. Several biodegradable materials have been investigated in the development of protein delivery systems, such as matrix, micro-structural, or nano-structural forms [8]. For intracellular protein transport, nano-scale carriers show high potential for the efficient delivery of proteins [9]. The proteins may be loaded into nanocarriers using different strategies, including direct conjugation via either chemical or genetic modifications, physical adsorption, and covalent/non-covalent encapsulation, which are dependent on the nanocarrier properties [9]. Subsequently, nanocarriers may interact with and function through the endocytosis pathway to enter target cells; thus, the delivery vehicle must be able to escape from the endosomal pathway to avoid lysosomal protein degradation [10]. Therefore, a suitable and effective intracellular protein delivery system is critical for protein drug delivery.

Gelatin is a natural, biodegradable, and bio-compatible as well as “generally regarded as safe” material by the U.S. Food and Drug Administration for use in pharmaceuticals, cosmetics, and food products. Gelatin nanoparticles (GNPs) have been used for drug delivery of doxorubicin, resveratrol, cycloheximide, and other drugs for tumor therapy [11,12,13]. Some extracellular active cytokines and growth factors, such as insulin, bone morphogenetic protein-2, and tissue-type plasminogen activator, were successfully encapsulated into GNPs and retained their bioactivity [14,15,16]. In our previous study, we showed that polyethyleneimine (PEI)-modified GNP could deliver DNA/plasmid with high efficiency in vivo and in vitro [17]. PEI is regarded as the most effective cationic polymer for gene delivery since it was first used in 1995 [18]. PEI is composed of rich chemically reactive amino groups. Thus, PEI can electrostatically interact with negatively charged biomolecules including proteins, drugs, and nucleic acids to form polyelectrolyte complexes [19]. Furthermore, the buffer capacity of PEI can induce endosome osmotic swelling and destabilize the endosome membrane, enabling PEI-associated compounds to escape degradation within the acidic endosomal environment. Additionally, the high pH-buffering capacity of PEI can protect and stabilize the delivered molecules in the endosome and lysosome [20].

In this study, a nanoparticle-based protein delivery system was developed for applications in protein drug delivery. Gelatin nanoparticles were prepared by a modified desolvation method and grafted with 1.8-kD branched PEI by cross-linking the amino group of PEI. Characteristics such as low cytotoxicity, outstanding protein loading ability, and high efficiency of intracellular protein delivery demonstrated that the formulation designed in this study is a promising functional vehicle for therapeutic applications.

## 2. Materials and Methods

### 2.1. Chemicals

The main materials used in this study were listed below: type B Gelatin from bovine skin (type B) (G9382, Sigma, St. Louis, MO, USA), Polyethyleneimine (PEI, MW 1.8 kD) (167-17811, Wako, Osaka, Japan), Acteone (AC0061, Seedchem Company, Melbourne, Australia), Glutaraldehyde (25%) (073-00536, Wako, Japan), 1-ethyl-3-(3-dimethylaminopropyl) carbodiimide (EDC) (E6383, Sigma, St. Louis, MO, USA), Rhodamine B isothiocyanate (RITC) (R1755, Sigma, St. Louis, MO, USA ), Albumin from bovine serum (BSA) (A2153, Sigma, St. Louis, MO, USA), Albumin–fluorescein isothiocyanate conjugate (BSA-FITC) (A9771, Sigma, St. Louis, MO, USA).

### 2.2. Synthesis of Gelatin Nanoparticles (GNPs), Gelatin Rhodamine B isothiocyanate Nanoparticles (GR NPs) and Gelatin Rhodamine B isothiocyanate Polyethyleneimine Nanoparticles (GR-PEI NPs)

Gelatin nanoparticles were synthesized by two-step desolvation process. Briefly, gelatin type B (Gelatin Type B from bovine skin, about 225 bloom, 50–100 kD, Sigma, St. Louis, MO, USA) was dissolved in distilled water at 40 °C and modified the concentration to 5% (*w*/*v*). Under the condition of stirring at 600 rpm and 40 °C, the same volume of acetone (Seedchem Company, Melbourne, Australia) was added into gelatin solution. At this time, the solution changed to milky, and precipitation started to extract. Stopping stirring and placing the gelatin-acetone mixture for 5 min. Supernatant was removed and distilled water was added to re-dissolve the extracted gelatin at 40 °C. Here, 1 mL gelatin solution was taken into 15 mL tube and freeze drying to measure the concentration of gelatin solution. In the second step, gelatin nanoparticles were synthesized by adding drop-wise an excess of acetone into gelatin solution and cross-linked with glutaraldehyde (Wako, Japan). The concentration of gelatin solution was modified to 10 mg/mL and pH was adjusted to 2. The gelatin solution was heated to 40 °C and stirred with 1000 rpm on the hot plate. Then, acetone was drop-wise added into gelatin solution. When the volume ratio of gelatin versus acetone reached 1:3, GNPs may be extracted gradually. Glutaraldehyde was added, and the final concentration was 0.125%. Acetone was evaporated by stirring overnight. Then nanoparticles were harvested by centrifugation at 13,500 rpm for 30 min three times and re-dispersed in distilled water. Rhodamine B isothiocyanate (RITC) was conjugated in gelatin nanoparticles to generate GR-NP. Before conjugation, RITC was dissolved in Dimethyl sulfoxide (DMSO) and mixed with 1-ethyl-3-(3-dimethylaminopropyl) carbodiimide (EDC) (Sigma, St. Louis, Missouri, USA) on equal molar ratio. After 30 min interaction of carboxyl group of RITC with EDC, the suspension of GNPs was added into RITC solution which had been reacted with EDC (weight ratio of GNPs:RITC = 1000:1). After stirring for 4 h, the final suspension was then dialyzed against distilled water using a dialysis membrane (MWCO: 12–14 kDa, Spectrum Laboratories, Rancho Dominguez, CA, USA) for 24 h to remove the un-reacted molecules. GR-PEI NPs were generated by the conjugation of PEI to GR NPs. In briefly, at first, the pH value of GR NP suspension was adjusted to acidic condition. Then 1.8-kD PEI was added drop-wise into the GR NP suspension under stirring at 1000 rpm at room temperature and the pH value was adjusted under 5. Subsequently, EDC was added into the suspension to final concentration of 5 mM. After 4 h reaction, the conjugated nanoparticles were dialyzed against distilled water using a dialysis membrane for 24 h to remove the un-reacted molecules.

### 2.3. Particle Size and Zeta Potential

The hydrodynamic diameters and zeta potentials of GR NPs and GR-PEI NPs were determined by photon correlation spectroscopy. Diameter measurements were obtained with dynamic laser light scattering (Zetasizer Nano ZS90, Worcestershire, UK) using He-Ne ion laser (633 nm) as the incident beam. Data was obtained at a detection angle of 90° at 25 °C and analyzed by a cumulant method to calculate the hydrodynamic diameters. The zeta-potentials of the NPs were evaluated by the laser-doppler electrophoresis method using Zetasizer Nano ZS90. The zeta potential was measured at a detection angle of 90° at 25 °C. The results were expressed as mean values (±SEM) of three experiments.

### 2.4. Particle Morphology

Morphology of GR NPs and GR-PEI NPs was observed by Philips Tecnai F30 Field Emission Gun Transmission Micro-scope (FEG-TEM, Amsterdam, The Netherland). 2 μL of each NP sample was dropped on the copper TEM grid and air-dried overnight to make sure the dry condition of TEM grid. The morphology data of GR NPs and GR-PEI NPs were acquired by TEM (300 kV). The surface morphology of NPs was examined by MFP-3D-BIO Atomic Force Microscopy (Asylum Research, Santa Barbara, CA, USA). Each sample was prepared by depositing on a freshly cleaved mica substrate and dried under vacuum. A cantilever (AC240, Olympus, Osaka, Japan) was operated at tapping mode with scanning speed set at 1.5 Hz. The images were recorded at a 2 m/s linear scanning speed.

### 2.5. Amino Group Quantification

Sodium 2,4,6-trinitro-benzene-1-sulfonate (TNBS) (Sigma, St. Louis, Missouri, USA) was used to determine the amino group content. GR NPs and GR-PEI NPs samples were diluted to adequate concentration. 20 μL of each sample was mixed with 150 μL distilled water and 850 μL of TNBS working solution at pH 8.5 (1 mM TNBS and 0.1 M sodium bicarbonate) for 1 h at 37 °C. Absorbance at 420 nm was then measured by a microplate reader (Bio-Rad 550, Hercules, CA, USA). The free amino group content of the nanoparticles was determined with the glycine as standard.

### 2.6. Stability of GR NPs and GR-PEI NPs

Temperature stability of GR NPs and GR-PEI NPs was determined by being suspended in phosphate buffered saline (PBS). The above suspensions were then incubated at different temperatures of 50–100 °C for 10 min. After incubation, the diameter of each sample was determined by Zetasizer Nano ZS90 (Malvern, Worcestershire, UK). GR NPs or GR-PEI NPs were suspended and incubated for 30 min in aqueous condition with different pH (pH = 3–9) to investigate the pH stability of NPs. After incubation, the diameter and zeta potential were determined by Zetasizer Nano ZS90.

### 2.7. Cytotoxicity Analysis

3T3 fibroblast cells were cultured with the complete medium containing Dulbecco’s modified Eagle medium (DMEM) (Gibco, Paris, France) supplemented with 10% fetal bovine serum (FBS) and 1% streptomycin and penicillin. Cells were seeded with density of 5 × 10^3^ cells per well in a 48-well plate and incubated overnight at 37 °C with humidified 5% CO_2_ condition. Different concentration of GR NPs and GR-PEI NPs were added into wells and incubated with cells for different time point of 1–3 days. Cytotoxicity was analyzed by Cell Titer 96 Aqueous One Solution Cell Proliferation Assay (Promega, Madison, WI, USA) according to manufacturer’s protocol. In brief, cells were pre-washed with PBS and replaced with 200 μL of fresh complete medium. 20 μL of 3-(4,5-dimethylthiazol-2-yl)-5-(3-carboxymethoxyphenyl)-2-(4-sulfophenyl)-2*H*-tetrazolium (MTS) was then pipetted into each well followed by incubation at 37 °C for additional 2 h. The viable cells were immediately determined by measuring the absorbance at 490 nm using a 96-well plate reader. The percentage of cell viability was calculated by comparing the absorbance of control samples with absence of NPs.

### 2.8. Cell Lines

Murine 3T3 fibroblast cells (ATCC), Human Huh7 hepatoma cells (JCRB), HEK293 cells (ATCC), HS68 cells (BCRC, Hsinchu, Taiwan) were cultured with the complete medium containing Dulbecco’s modified Eagle medium (DMEM) (Gibco, Paris, France) supplemented with 10% fetal bovine serum (FBS) and 1% streptomycin and penicillin. Murine CT26 colon adenocarcinoma cells (ATCC) were cultured with complete medium containing Roswell Park Memorial Institute (RPMI) 1640 medium (Gibco, Paris, France) supplemented with 10% fetal bovine serum (FBS) and 1% streptomycin and penicillin.

### 2.9. Cellular Uptake Analysis

Cells were seeded in 6-well culture plate with the density of 2 × 10^5^ cells per well. After culturing overnight, different concentrations of GR NPs and GR-PEI NPs were added into the wells (final concentration were 0.5, 1, 5, 10, 50, 100 μg/mL). After incubation for 24 h, the cells were trypsinized and fixed with 4% paraformaldehyde solution for 10 min followed by PBS washing. The cellular uptake efficiency was evaluated by BD FACSCanto II flow cytometer (BD Bioscience, San Jose, CA, USA).

### 2.10. Fluorescent Microscopic Imaging

In vitro Fluorescent images of GR NPs and GR-PEI NPs were observed by fluorescent microscope. The candidate cells were incubated in 6-well culture plates with different concentrations of GR NPs and GR-PEI NPs (5, 10, 25 μg/mL) for 24 h at 37 °C. The cells were washed with PBS three times and fixed with 4% paraformaldehyde solution for 10 min. The nuclei were stained with 4′,6-diamidino-2-phenylindole (DAPI), a blue fluorescent dye, for 5 min and washed three times with PBS [21]. All the samples were observed by fluorescent microscope.

### 2.11. Analysis of Protein Binding Efficiency

To obtain the maximum binding efficiency, the weight ratio of NP versus protein was set as 4:1, 2:1, 1:1, and 1:2. GR NPs and GR-PEI NPs were prepared as working condition of 1 mg/mL in aqueous suspension with neutral pH value. Different amount of BSA was dissolved in distilled water for binding reaction. NP suspension was dropped into BSA solution under the strongly vortex. After 1 h reaction time, NP-protein complexes were separated by 13,500 rpm centrifugation for 15 min. The supernatant was collected to evaluate the residual BSA by BCA protein assay kit (Pierce, Waltham, MA, USA).

### 2.12. Intracellular Protein Delivery of GR-PEI NPs

10 μg GR-PEI NPs binding 1 μg BSA-FITC or 20 μg GR-PEI NPs binding 1 μg BSA-FITC to evaluate the intracellular protein delivery efficiency. To prepare the BSA-FITC and GFP protein loading GR-PEI NPs, the equal volume of GR-PEI NP suspension and the proteins with the weight ratio of 10 mg:1 mg were mixed with strong vortex. The flow cytomerty data and laser scanning confocal microscopy (Olympus fv300, Osaka, Japan) images were used to evaluate the protein delivery efficiency subsequently. pcDNA-Rab7-HA was transfected into HEK293 with Jet-prime transfection reagent (Polyplus, New York, NY, USA) for endosome tracker. Fluorescent intensity was analyzed by ImageJ (NIH Image, Bethesda, MD, USA).

### 2.13. In Vivo Accumulation of GR-PEI NPs in Tumor Tissues

Female BALB/cAnN.Cg-Foxn1nu/CrlNarl nude mice at 5–8 weeks of age were purchased from the Animal Center, National Laboratory, Taipei, Taiwan). The experiments were performed according to the guidelines of the institutional animal care and use committee of National Taiwan University (NTU-100-EL-109). The xenograft tumors of C26 cells were established and used to verify the local accumulating ability of nanoparticles designed in this study. For solid tumor formation, 2 × 10^6^ C26 cells were suspended in PBS and injected subcutaneously into the flank of 6-week-old female BALB/c Nude mice, respectively. The respective tumor volume measurement was based on the following formula: tumor volume = (major axis) × (minor axis)^2^ × (π/6). When the tumor size reached 200 mm^3^, 40 μg GR-PEI NPs in PBS were administrated in tumor mice via intra-tumor injection. After injection 72 h, the accumulation condition of nanoparticles in tumors was evaluated. Solid tumors were harvested and embedded in OCT under freezing temperature. Cryo-sections were obtained by Leica CM-3050S Cyrostats (Leica, Wetzlar, Germany) with the thickness of 12 μm. The fluorescent signals in tumor cryo-sections were observed by fluorescent microscopy.

### 2.14. Statistics

Figure data in the bar charts represent means ± SEM and were obtained from average data of three independent experiments. Statistical significance was calculated using a two-tailed Student’s-test. Differences with the *p* value of less than 0.05 were considered significant, and those with P value of less than 0.01 were considered really significant.

## 3. Results

### 3.1. Physical Characteristics and Morphology of GR NPs and GR-PEI NPs

Through 1-ethyl-3-(3-dimethylaminopropyl)-carbodiimide (EDC) cross-linking, cationic PEI was modified on the surface of GR NPs. The average hydrodynamic diameter and zeta potential of GR NPs were measured by photon correlation spectroscopy.

**Figure 1:**
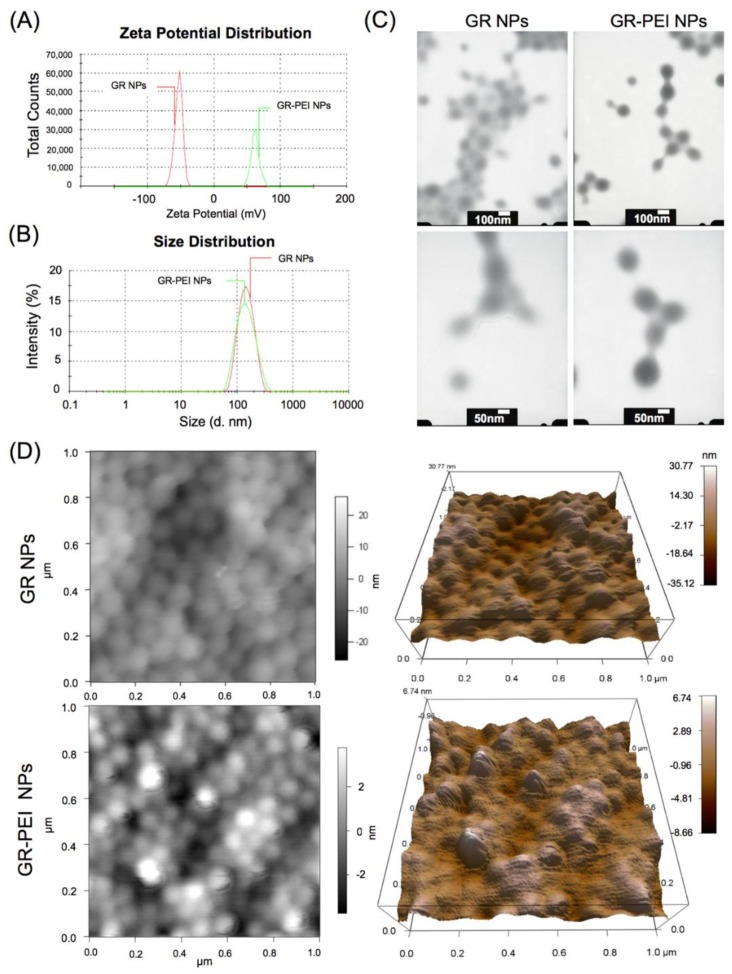
Physical characteristics of GR NPs and GR-PEI NPs. (**A**,**B**) The particle size of GR NPs and GR-PEI NPs showed the similar size distribution, but the zeta potential of GR NPs and GR-PEI NPs was different in neutral condition. (**C**) TEM images of GR NPs and GR-PEI NPs showed the spherical morphology by the preparation protocol in this study (upper panel: scale bar = 100 nm; lower panel: scale bar = 50 nm). (**D**) AFM images demonstrated the uniform and spherical morphology after surface modification.

As shown in Table 1 and Figure 1A, GR NPs showed a negative charge at −53.2 mV. After surface modification by 1.8-kD PEI, the zeta potential of GR-PEI NPs was approximately +53.9 mV. After conjugation with rhodamine B isothiocyanate (RITC), the size of GR NPs was slightly increased to approximately 111.8 nm with a polydispersity index of 0.175, and the particle size was approximately 109.3 nm after surface modification with 1.8-kD PEI (Table 1 and Figure 1B).

The surface morphology of NPs with different formulations was observed by atomic force microscopy. The surface of GR NPs showed a smooth pattern, and the surface remained smooth after surface modification with PEI (Figure 1C,D).

### 3.2. Particle Size and Zeta Potentials of GR NPs and GR-PEI NPs in Different pH and Temperture Conditions

**Figure 2:**
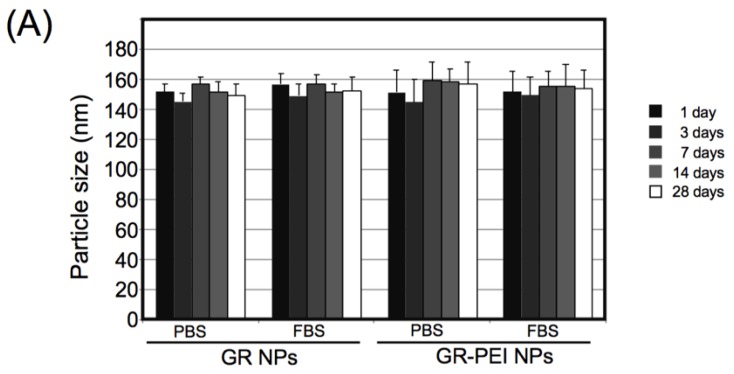

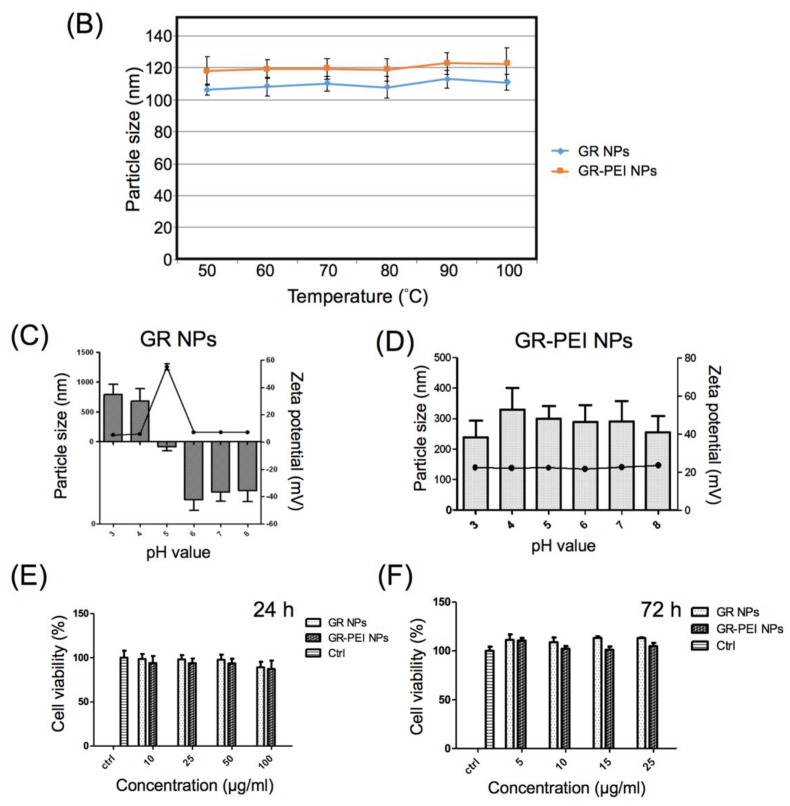
The stability of GR NPs and GR-PEI NPs. GR NPs and GR-PEI NPs were incubated in PBS and PBS with 10% fetal bovine serum (FBS) at 37 °C for up to 28 days (**A**) and heated up to 100 °C (**B**). The particle size (bar) and zeta potential (line) of GR NPs (**C**) and GR-PEI NPs (**D**) were detected in the different pH value conditions. (**E**) The cytotoxicity assay of GR NPs and GR-PEI NPs. Cytotoxicity of excess GR NPs and GR-PEI NPs (up to 100 μg/mL) was evaluated. Each formulation was incubated with 3T3 fibroblast cells for 24 h followed by MTS assay analysis. (**F**) The relative cell viability of 3T3 fibroblast cells incubated with GR NPs and GR-PEI NPs for 72 h. The representative results are shown as M ± SD (*n* = 3). Each bar represents the means of three determinations ±SD.

To determine the physical properties of GR NPs, the particle sizes and zeta potentials were measured under different temperatures, and pH values. As shown in Figure 2A, the particle size did not significantly change after incubation at 37 °C in the buffer for up to 28 days. GR NPs and GR-PEI NPs were heated from 50 to 100 °C and the particle size variation was determined after heating (Figure 2B). The results revealed no significant changes in the size distribution of both nanoparticles from 50 to 100 °C (Figure 2B). The results demonstrated that glutaraldehyde (GA) cross-linking strongly stabilized the gelatin nanoparticles.

The pH stability of nanoparticles was determined. We found that the particle sizes of GR NPs were 132 ± 6.65 and 160 ± 4 nm at below and above pH 5, respectively. However, the particle size increased to 1258 ± 50 nm at pH 5, indicating the aggregation of GR NPs at this pH (Figure 2C). In addition, the surface charge of GR NPs was positive below pH 4. Under neutral conditions, the surface charge of GR NPs became negative (up to −42.3 ± 7.66 mV) (Figure 2C). In contrast, the particle sizes of GR-PEI NPs were 139 ± 0.87, 140 ± 4.3, and 146 ± 1.09 nm under acidic, neutral, and basic conditions, respectively, demonstrating that particle sizes were similar under different pH conditions (Figure 2D). The surface charges of GR-PEI NPs were also similar and remained positive at different pH values (Figure 2D). These results reveal that surface modification of PEI contributed to the stability of gelatin nanoparticles under various pH conditions.

### 3.3. Cytotoxicity Analysis

To investigate the cytotoxic effects of GR NPs, 3T3 fibroblast cells were incubated with different concentrations of GR/GR-PEI NPs for 24 h, and cell viability was measured by MTT assay. As shown in Figure 2E, no significant cytotoxicity of GR and GR-PEI NPs (each from 10 to 100 μg/mL) was observed. For 48 and 72 h of long-term incubation, no significant cytotoxicity of GR/GR-PEI NPs was observed on fibroblast cells (Figure 2F).

### 3.4. Protein Binding Efficiency and Cellular Uptake of GR NPs and GR-PEI NPs

**Figure 3:**
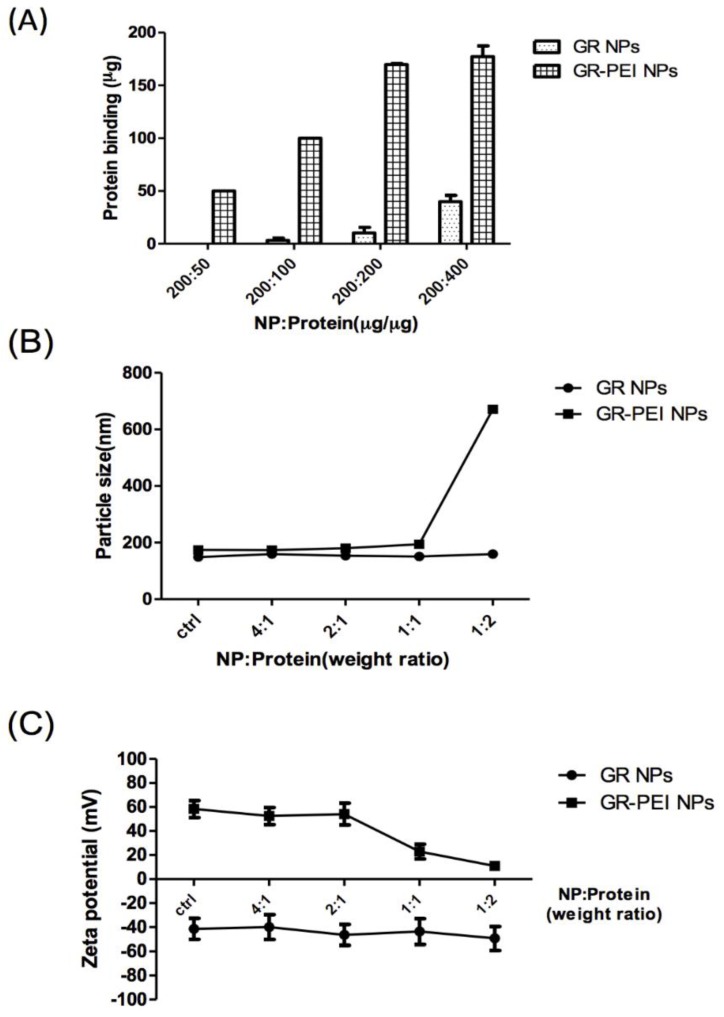
Protein binding efficiency of GR NPs and GR-PEI NPs. (**A**) Protein binding ability was determined with different NP:Protein ratio. (**B**,**C**) Different weight ratio of bovine serum albumin (BSA) was interacted with GR NPs or GR-PEI NPs for 1 h. The variation of particle size and zeta potential were measured by DLS spectroscopy. The polydispersity index (PDI) of each group was less than 0.1. The representative results are shown as M ± SD (*n* = 3). Each bar represents the means of three determinations ±SD.

Bovine serum albumin (BSA) was used as a model protein in this study. BSA had negative charge under the neutral condition and was bound by GR NPs and GR-PEI NPs via electric adsorption. As shown in Figure 3A, GR-PEI NPs possessed more remarkable protein binding efficiency than GR NPs. The 100% binding efficiency was reached at weight ratios of both 200:50 and 200:100 (NP:protein). Moreover, we examined the particle size and zeta potential of each type of nanoparticle loaded with model protein to determine the effects of protein loading. The results showed that the particle size of GR-PEI NPs after protein loading was relatively stable, whereas that of GR-PEI NPs was increased when the NP/protein weight ratio was less than 1 (Figure 3B). The surface charge of GR NPs remained negative after BSA binding. The zeta potential of GR-PEI NP began decreasing when the NP/protein weight ratio was equal to or less than 1 (Figure 3C).

### 3.5. Cellular Uptake of GR NPs and GR-PEI NPs

**Figure 4:**
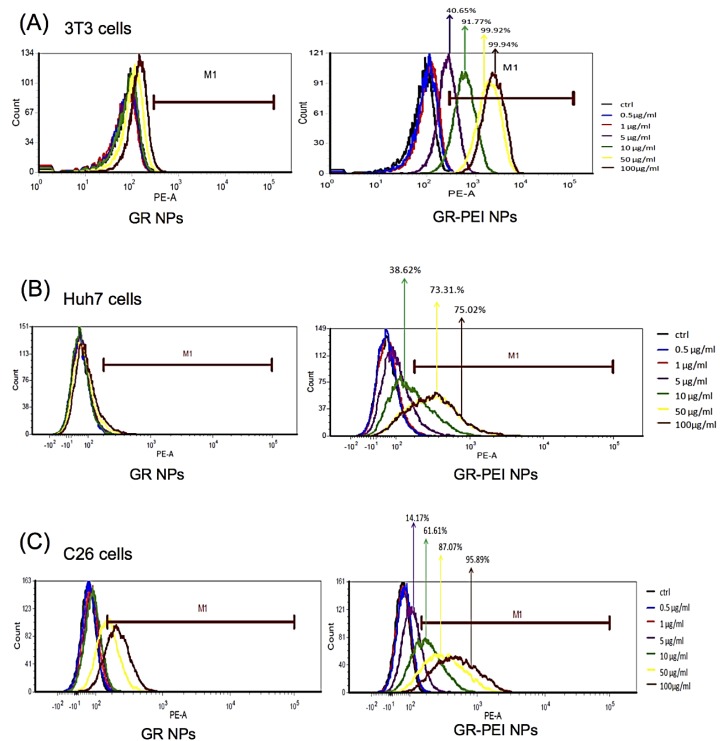
Cellular uptake of NPs in different cells. Cellular uptake analysis of 3T3 cells (**A**), Huh7 cells (**B**) and C26 cells (**C**) by flow cytometry. The cells were treated with different concentrations of GR NPs or GR-PEI NPs for 24 h. The cellular uptake efficiency was assayed by quantifying the percentage of rhodamine B isothiocyanate (RITC) positive cells.

3T3 fibroblasts, human Huh7 hepatoma cells, and murine C26 colon adenocarcinoma cells were used to estimate the cellular uptake efficiency of RITC-labeled NPs. Cells with the RITC signals were quantified by flow cytometry. 91.77% of 3T3 cells acquired GR-PEI NPs when treated with 10 μg/mL of GR-PEI NPs (Figure 4A), 73.31% of Huh7 cells acquired GR-PEI NPs when treated with 50 μg/mL of GR-PEI NPs (Figure 4B), and 95.89% of C26 cells acquired GR-PEI NPs when treated with 100 μg/mL of GR-PEI NPs (Figure 4C).

### 3.6. Intracellular Protein Delivery of GR-PEI NPs

**Figure 5:**
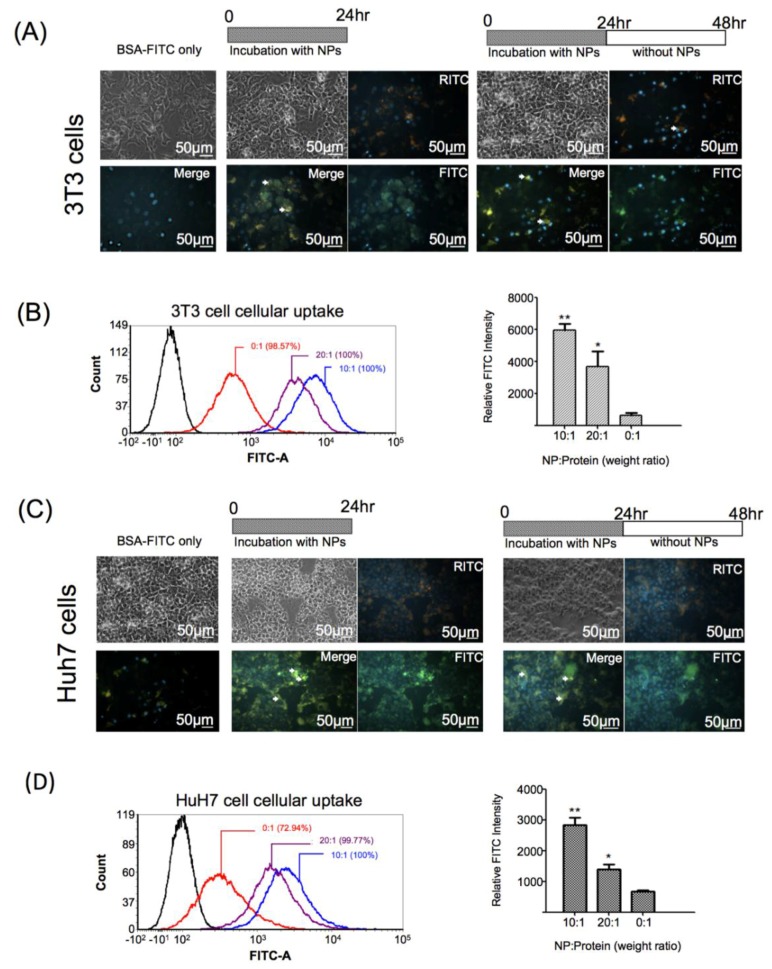
Intracellular protein delivery by GR-PEI NPs. Fluorescent images of protein delivery by GR-PEI NPs in 3T3 fibroblast (**A**) and Huh7 cells (**C**) for 24 h and 24 h after treatment (Bar: 50 μm). The cells were incubated with BSA-FITC carried by GR-PEI NPs and free BSA-FITC (final protein concentration was 5 μg/mL). Different formulations of BSA-FITC loaded GR-PEI NPs and free BSA-FITC (final protein concentration was 2 μg/mL) were added to 3T3 fibroblast cells and Huh7 cells for 24 h. The protein delivery efficiency was evaluated by flow cytometry and quantified by measuring the geometric mean of FITC fluorescent intensity (**B**,**D**). The representative results are shown as M ± SD (*n* = 3). Each bar represents the means of three determinations ±SD. * *p* < 0.05, ** *p* < 0.01 among the indicated groups.

BSA-FITC-loaded GR-PEI NPs were used to determine the intracellular delivery efficiency of proteins in 3T3 and Huh7 cells. Cells were incubated with two formulations of BSA-FITC-loaded GR-PEI NPs (weight ratios of 10:1 and 20:1) at several time points. Incubation with free BSA-FITC was used as the control group to compare the intracellular delivery efficiency of proteins with or without carrier. After incubation for 24 h, considerable uptake of nanoparticles in both cell lines was observed (middle panel of Figure 5A,C). Compared to the control group, higher FITC intensity was observed in both cell lines treated with GR-PEI NPs of two different formulations (Figure 5B,D). As shown in Figure 5A,C, BSA molecules were released from GR-PEI NPs in the cells after 24 h of incubation. Additionally, more BSA protein was released from the particles in 3T3 and Huh7 cells after more 24-h culture (Right panel of Figure 5A,C), indicating the protein delivery capacity of GR-PEI NPs.

**Figure 6:**
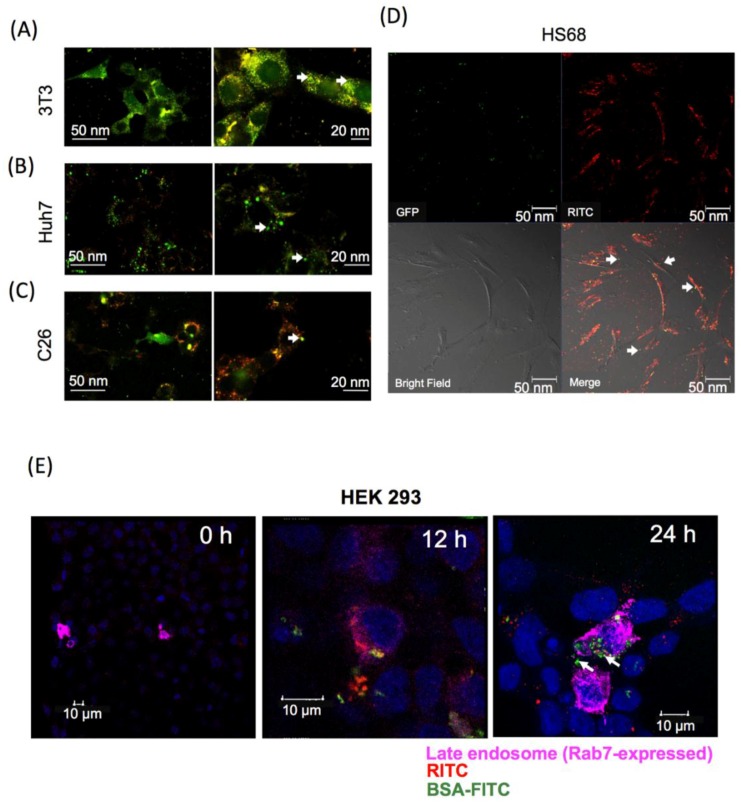
Intracellular protein release from GR-PEI NPs. Confocal laser microscopic images of intracellular protein release. The tomography of cells by confocal laser microscope demonstrated the intracellular BSA-FITC was released from GR-PEI NPs in 3T3 fibroblast cells (**A**), Huh7 hepatoma cells (**B**), C26 colon adenocarcinoma cells (**C**). GFP was released from HS68 fibroblast cells (**D**). Rab7 was used as late endosome tracker. The sub-cellular localization of BSA-FITC and GR-PEI NPs was observed in HEK293 cells that were transfected with late endosome marker, Rab7, (Bar: 10 μm) (**E**). GR-PEI NPs were shown as red signals; BSA-FITC and GFP were shown as green signals; Endosome marker Rab7 was shown as purple pink signals (Alexa Fluor 647, Thermo Fisher, Waltham, MA, USA). Co-localization of GR-PEI NPs and BSA-FITC was shown in yellow.

Confocal laser microscopy was used to detect the released intracellular proteins from GR-PEI NPs. The results showed green fluorescence from BSA-FITC throughout the cytoplasm with some red fluorescence from GR-PEI NPs, demonstrating that most BSA-FITC was released from GR-PEI NPs after 24 h incubation in 3T3, Huh7, and C26 cells (Figure 6A–C). The stability of protein structure is important for protein activity. We delivered functional green fluorescent protein (GFP) into human HS68 fibroblast cells by into GR-PEI NPs. GFP could be observed in the cytosol of human HS68 cells (Figure 6D). In addition, BSA-FITC with GR-PEI NPs could escape from late endosomes and could be released into cytosol at 24 h after delivery (Figure 6E). These data indicated that GR-PEI NPs increased the efficiency of protein delivery.

### 3.7. In Vivo Accumulation of GR-PEI NPs

**Figure 7:**
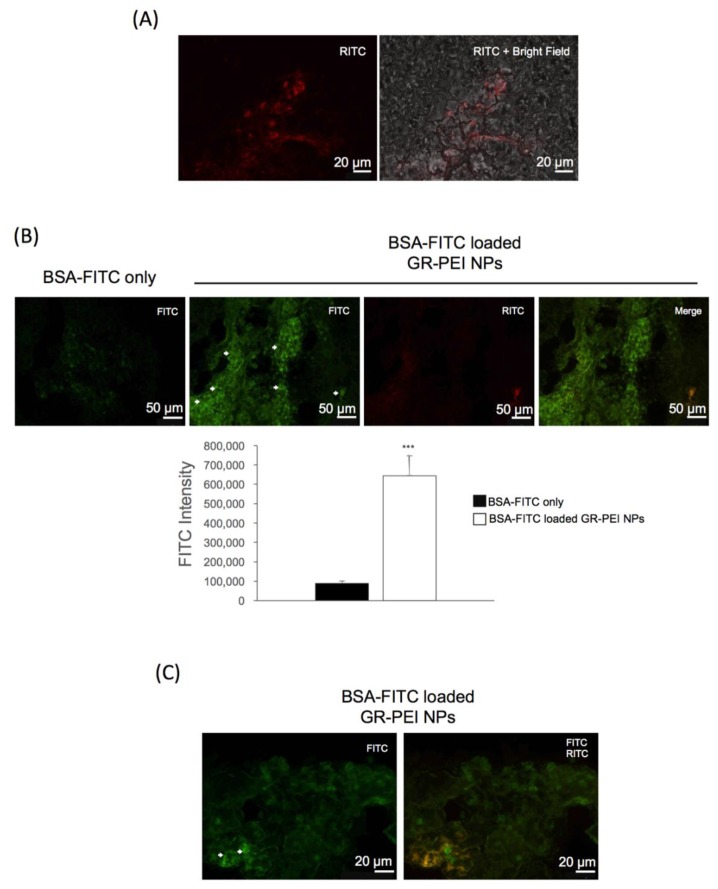
In vivo protein delivery by GR-PEI NPs. (**A**) 40 μg GR-PEI NPs were administrated in C26 tumor-bearing mouse by intra-tumor injection for 72 h (*n* = 3). The RITC fluorescence in cryo-section (12 μm) of C26 solid tumor was observed by fluorescent microscope. The 40X images showed the cellular uptake of GR-PEI NPs in the tumor tissue. (**B**) BSA-FITC loaded GR-PEI NPs with weight ratio of 10:1 was used to evaluate the protein delivering efficiency of GR-PEI NPs in vivo. BSA-FITC loaded GR-PEI NPs were administrated in C26 cells tumor mouse model by intra-tumor injection for 72 h (*n* = 3). Fluorescent microscopic image was used to evaluate the protein delivery in tumor tissues. The figures showed the intra-tumor accumulation of GR-PEI NPs and the cellular uptake of BSA-FITC. Free BSA-FITC delivery was showed in left panel. (Bar: 50 μm) Each bar represents the means of three determinations ±SD. *** *p* < 0.001 among the indicated groups compared with control group. (**C**) Intracellular delivery of BSA-FITC was shown as the spherical green fluorescent signals. (Bar: 20 μm).

To determine whether GR-PEI NPs can accumulate in vivo, GR-PEI NPs were administered at the tumor site of C26 tumor-bearing mice for 72 h. As shown in Figure 7A, RITC signals were detected in the tumor tissue. The uptake and delivery of BSA-FITC by GR-PEI NPs was also observed in fluorescent images. Compared to the control group, the tissue sections administered BSA-FITC loaded GR-PEI NPs showed significantly more green fluorescence in the center of the tumor with spherical morphological signals (Figure 7B,C).

## 4. Discussion

**Figure 8:**
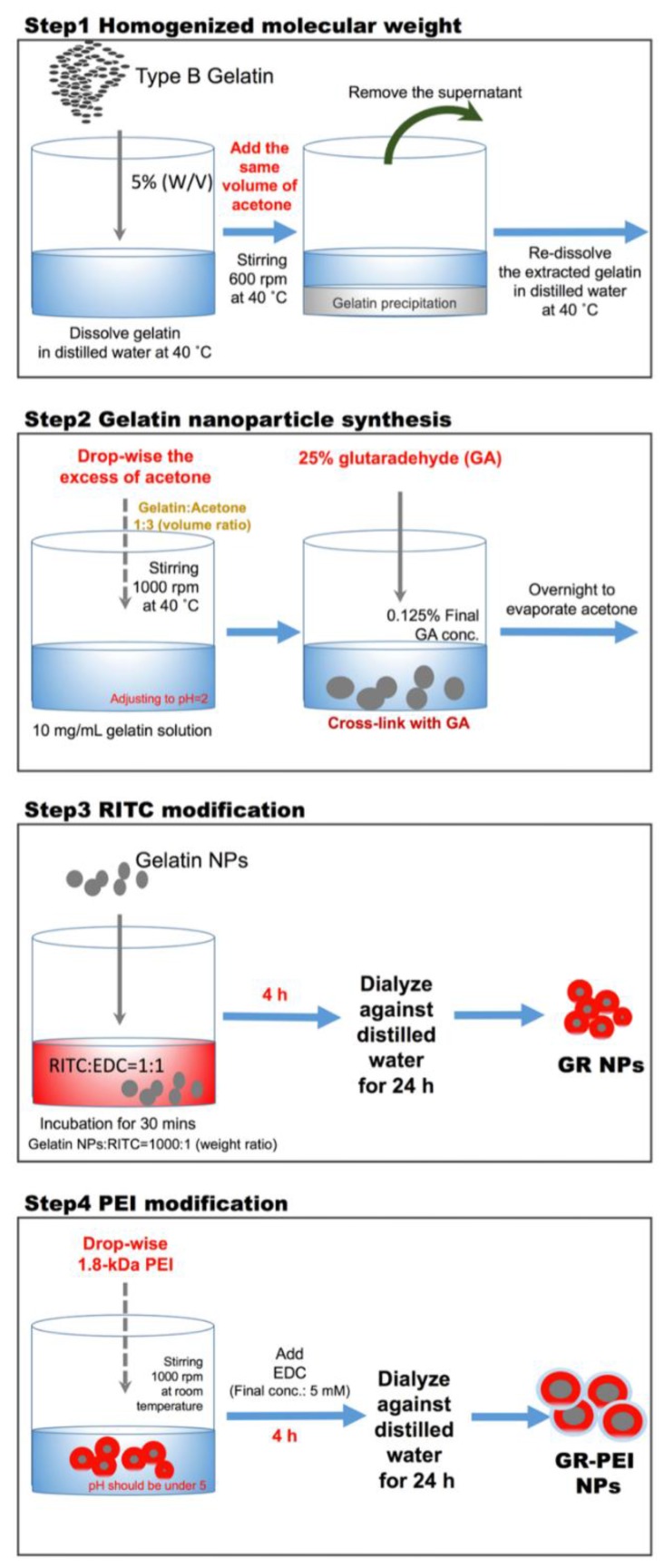
Scheme of particle synthesis.

Drug delivery by nanocarriers is advantageous for delivery efficiency and specificity. Nowadays, intracellular functional protein delivery by nano/micro carriers has become more important for biomedical application, such as cell reprogramming in regenerative medicine, cell fate transition, and cancer differentiation therapy. Here, we generated cationic gelatin nanoparticles, GR-PEI NPs formulated from type B gelatin, followed by surface modification of 1.8-kD branched PEI for intracellular delivery (Figure 8). Our results showed that the nano-size, spherical morphology, low cytotoxicity and stability of the formulated carriers provided appropriate characteristics for the nanocarriers for intracellular delivery of protein drugs. GR-PEI NPs showed high protein loading rates and good cellular internalization of protein. Additionally, intra-tumor administration of protein-loaded GR-PEI NPs revealed the uptake efficiency in tumor tissues, suggesting the potential of in vivo administration of protein drugs in the future applications.

Gelatin is a biocompatible and biodegradable material that has been extensively applied in biomedical research [22]. Gelatin nanoparticles show hydrogel like characteristics even after glutaraldehyde cross-linking [23]. Previous studies reported that the particle sizes of gelatin nanoparticles were typically 200–400 nm. Gelatin nanoparticles less than 200 nm showed high cellular uptake by tumor cells via enhanced permeability and retention effects because of the leakiness of the tumor vasculature [24]. Enhanced cellular uptake can induce higher drug accumulation in tumor tissues and improve pharmacological efficacy. In this study, our protocol using the modified formulation generated gelatin nanoparticles of less than 150 nm. Besides, atomic force microscopy and transmission electron microscopy images of our generated gelatin nanoparticles revealed uniform morphologies, spherical and smooth surface. These properties also increase the cellular uptake, preventing the circulation clearance.

Some researches indicated that positively charged nanoparticles showed the greatest efficiency in cell-membrane penetration and cellular internalization [25]. However, inappropriate cationic components on the NPs might interact with proteoglycans on the cell surface, which may trigger cellular necrosis and apoptosis [26,27,28,29]. Thus, the degree of cationic surface modification must be evaluated to balance the enhancement of cellular internalization and cytotoxicity. Among the cationic polymers, PEI has been reported as an endosomal escape agent due to its pH-buffering capacity. It promotes burst-release from the endosome resulting from increasing osmotic pressure and may prevent drug or bio-molecules from lysosomal digestion [30]. Our previous study showed 10-kD PEI was more cytotoxic while lower molecular weight PEI exhibited lower colloidal stability and electric binding efficiency [17]. Therefore, the formulation of gelatin nanoparticles with surface modification of 1.8-kD PEI was the most suitable and used in this study. Our results showed there was no significant difference in cell viability between treated GR NPs and GR-PEI NPs at high concentrations (up to 100 μg/mL) (Figure 2E). The result revealed low cytotoxicity of the nanoparticles, indicating that this formulation of PEI surface modified gelatin nanoparticles may be biocompatible for in vitro and in vivo applications. Cationic surface-modified nanoparticles were investigated for intracellular gene delivery or siRNA delivery. Through electrostatic interactions, a negatively charged compound can be adsorbed on the cationic surface of nanoparticles. It is thought that the branched structure of PEI provides a physical barrier to protect plasmid DNA or siRNA against nuclease digestion [17,31]. The strong electrostatic interactions between PEI and negatively charged protein may make GR-PEI NPs good protein carriers and protect the protein from degradation by environmental factors.

Proteins are biological amphoteric molecules containing both acidic and basic functional groups. At a pH below the isoelectric point (IEP) of a protein, proteins carry a net positive charge. In contrast, proteins carry a net negative charge at a pH above the IEP. In this study, BSA with an IEP of 5.07 was used as the protein model. We hypothesized that BSA adsorbed on the GR-PEI NPs would be stable in a neutral physiological environment, but BSA may be released from GR-PEI NPs in the mildly acidic endosome because the pH value decreased below the IEP of BSA. This release profile may affect protein intracellular delivery by preventing proteins from being released before entry into cells. Intracellular delivery of protein by the GR-PEI NPs designed in this study was found to be effective. Fluorescent microscopic images and confocal images revealed the intracellular release of BSA-FITC, indicating that the protein was efficiently delivered into the cells by the nanocarriers and released in the cytoplasm. The results also demonstrated the endosomal escape ability of PEI. The confocal images indicated the separation signal of RITC and FITC, suggesting the release of protein from nanoparticles. Additionally, the fluorescent microscopic images revealed spreading of the FITC signal in whole cells, demonstrating that the GR-PEI NPs and BSA-FITC escaped from the endosome, avoiding lysosomal digestion.

Protein drug delivery is also used in the biomedical applications. An amine-terminated generation 5 polyamidoamine dendrimer was conjugated with guanidinobenzoic acid (GBA) for the protein vehicle [32]. Biodegradable Polycaprolactone/Maltodextrin (PCL-MD) nano-carrier was used to deliver proteins into LNCaP cells [33]. The high efficiency of intracellular protein delivery can be safely used in cell differentiation and reprogramming in regenerative medicine and the differentiation therapy of cancers. In the study, we developed a gelatin-based nanocarrier to deliver protein molecules. The result of in vivo analysis of protein delivery demonstrated the ability of intracellular protein delivery to the tumor site and the distribution of nanocarriers. These data indicate that protein delivery by GR-PEI NPs can be applied in protein-based drug delivery techniques. For further cell or tissue targeting, functional groups on GR-PEI NPs may be conjugated with specific ligands, such as antibodies, folate, or specific peptides to recognize specific receptors. Ligand-conjugated nanocarriers may provide active targeting functions to enhance the specificity in therapeutic efficacy with fewer side effects.

## Figures and Tables

**Table 1:** Physical properties of nanoparticles.

Nanoparticles	Particle Size (nm)	Polydispersity Index (PDI)	Zeta Potential (mV)	Amino Group Content (mM)
Gelatin	95.99 ± 0.267	0.133 ± 0.006	−50.6	0.077 ± 0.004
Gelatin-RITC (GR NPs)	111.8 ± 1.000	0.175 ± 0.012	−53.2	0.0263 ± 0.006
Gelatin-RITC-PEI (2:1) (GR-PEI NPs)	109.3 ± 0.458	0.142 ± 0.023	+53.9	0.290 ± 0.009
